# Explaining variable effects of an adaptable implementation package to promote evidence-based practice in primary care: a longitudinal process evaluation

**DOI:** 10.1186/s13012-021-01166-4

**Published:** 2022-01-27

**Authors:** Liz Glidewell, Cheryl Hunter, Vicky Ward, Rosemary R. C. McEachan, Rebecca Lawton, Thomas A. Willis, Suzanne Hartley, Michelle Collinson, Michael Holland, Amanda J. Farrin, Robbie Foy, on behalf of the ASPIRE programme team, Sarah Alderson, Paul Carder, Susan Clamp, Robert West, Martin Rathfelder, Claire Hulme, Judith Richardson, Tim Stokes, Ian Watt

**Affiliations:** 1 Leeds Institute of Health Sciences, University of Leeds, Leeds, England; 2 Hull York Medical School, University of York, York, England; 3 University of East London, London, England; 4 School of Management, University of St Andrews, St Andrews, Scotland; 5 Bradford Institute for Health Research, Bradford Teaching Hospitals NHS-Foundation Trust, Bradford, England; 6 School of Psychology, University of Leeds, Leeds, England; 7 Leeds Institute of Clinical Trials Research, University of Leeds, Leeds, England

**Keywords:** Tailored intervention, Adaptable implementation package, Theoretical Domains Framework, Normalization Process Theory, Process evaluation, Audit and feedback, Educational outreach, Computerised prompts, Clinical reminders, Primary care, Fidelity

## Abstract

**Background:**

Implementing evidence-based recommendations is challenging in UK primary care, especially given system pressures and multiple guideline recommendations competing for attention. Implementation packages that can be adapted and hence applied to target multiple guideline recommendations could offer efficiencies for recommendations with common barriers to achievement. We developed and evaluated a package of evidence-based interventions (audit and feedback, educational outreach and reminders) incorporating behaviour change techniques to target common barriers, in two pragmatic trials for four “high impact” indicators: risky prescribing; diabetes control; blood pressure control; and anticoagulation in atrial fibrillation. We observed a significant, cost-effective reduction in risky prescribing but there was insufficient evidence of effect on the other outcomes. We explored the impact of the implementation package on both social processes (Normalisation Process Theory; NPT) and hypothesised determinants of behaviour (Theoretical Domains Framework; TDF).

**Methods:**

We conducted a prospective multi-method process evaluation. Observational, administrative and interview data collection and analyses in eight primary care practices were guided by NPT and TDF. Survey data from trial and process evaluation practices explored fidelity.

**Results:**

We observed three main patterns of variation in how practices responded to the implementation package. First, in *integration and achievement*, the package “worked” when it was considered distinctive and feasible. Timely feedback directed at specific behaviours enabled continuous goal setting, action and review, which reinforced motivation and collective action. Second, *impacts on team-based determinants* were limited, particularly when the complexity of clinical actions impeded progress*.* Third, there were *delivery delays and unintended consequences*. Delays in scheduling outreach further reduced ownership and time for improvement. Repeated stagnant or declining feedback that did not reflect effort undermined engagement.

**Conclusions:**

Variable integration within practice routines and organisation of care, variable impacts on behavioural determinants, and delays in delivery and unintended consequences help explain the partial success of an adaptable package in primary care.

Contributions to the literature

- We drew upon the Theoretical Domains Framework (TDF) and Normalisation Process Theory (NPT) in a longitudinal study to explain the variable success of an adaptable implementation package promoting evidence-based primary care.
- The package worked best when it was sufficiently distinct from but could be integrated within existing organisational routines, with clear benefits for patients. It failed when delivery was delayed and professionals could not observe any improvement resulting from their efforts.
- This study demonstrates the value of integrating psychological and sociological perspectives to design implementation strategies. TDF enabled mapping of the implementation behaviours to attend to, and NPT generated an understanding of how these dynamically interwove with work allocation and negotiation.

## Background

Implementing any evidence-based practice within the constraints and competing priorities of United Kingdom primary care is difficult. Implementing numerous evidence-based practices from a wide range of clinical guidelines in this context is even more challenging [1]. Systematic reviews indicate a range of implementation strategies, such as audit and feedback and educational outreach, can enhance implementation [2–6]Studies typically focus on evaluating interventions for single clinical conditions (e.g. type 2 diabetes) or behaviours (e.g. antibiotic prescribing). This limits generalisability, or the confidence that an implementation strategy that works for one targeted problem will work for another [7]. There are insufficient resources to develop and evaluate interventions for each implementation problem separately.

We developed an implementation package for UK primary care with the aims of being adaptable for different clinical priorities and sustainable within existing resources. We selected four “high impact” quality indicators: risky prescribing (focused on non-steroidal anti-inflammatory drugs; NSAIDs); control of type 2 diabetes; blood pressure control in people at high risk of cardiovascular events; and anticoagulation for stroke prevention in atrial fibrillation (Table 1) [1]. We conducted interviews with primary care staff using the Theoretical Domains Framework (TDF) and identified a common set of determinants of adherence to these indicators [8]. We consulted with primary care stakeholders to develop an implementation package based upon evidence-based implementation techniques, such as audit and feedback, educational outreach, and computerised prompts and reminders. This implementation package incorporated behaviour change techniques tailored to the determinants identified in the interviews with primary care staff [8],with content adapted to each of the four indicators [9–11] (Table 2). Whilst indicators could not be completely independent of the intervention (e.g. given that feedback used the indicators), the interventions were designed so that indicators and related content could be dropped in.

**Table 1:** Clinical Indicators targeted by the intervention package

Clinical indicators	Description
Risky prescribing	Proportion of patients meeting at least one of nine indicators of high-risk NSAID and anti-platelet prescribing: prescription of a traditional oral NSAID or low-dose aspirin in patients with a history of peptic ulceration without co-prescription of gastro-protection; traditional oral NSAID in patients aged 75 years or over without co-prescription of gastro-protection; traditional oral NSAID and aspirin in patients aged 65 years or over without co-prescription of gastro-protection; aspirin and clopidogrel in patients aged 65 years or over without co-prescription of gastro-protection; warfarin and traditional oral NSAID; warfarin and low-dose aspirin or clopidogrel without co-prescription of gastro-protection; oral NSAID in patients with heart failure; oral NSAID in patients prescribed both a diuretic and an angiotensin-converting-enzyme inhibitor (ACE-I) or angiotensin receptor blocker (ARB); oral NSAID in patients with chronic kidney disease (CKD).
Diabetes	Proportion of patients with type 2 diabetes achieving all three treatment targets: BP below 140/80 mmHg (or 130/80 mmHg if kidney, eye or cerebrovascular damage); HbA1c value below or equal to 59 mmol/mol; cholesterol level below or equal to 5.0 mmol/l
Blood pressure	Proportion of patients achieving the lowest appropriate BP target: under 140/90 mmHg if aged under 80 years with hypertension, coronary heart disease, peripheral arterial disease, a history of stroke or transient ischemic attack, or a 10 year cardiovascular disease risk of 20% or higher; under 150/90 mmHg if aged 80 years and over with hypertension; under 140/80 mmHg if aged under 80 years with diabetes, under 130/80 mmHg if complications of diabetes or aged under 80 years with chronic kidney disease and proteinuria.
Anticoagulation	Combined proportion of men with AF and a CHA2DS2-VASc score of 1 and women with a CHA2DS2-VASc score of 2 or above prescribed anticoagulation therapy.

Abbreviations: *ACE*-I angiotensin-converting-enzyme inhibitor; *ARB* angiotensin receptor blocker; *BP* blood pressure; *CHA*_*2*_*DS*_*2*_*-VASc* congestive heart failure, hypertension, age ≥75, diabetes, stroke, vascular disease, age between 65 and 74, and female sex; *CKD* chronic kidney disease; *HbA1c* haemoglobin A1c; *NSAID* non-steroidal anti-inflammatory drug

**Table 2:** Intervention package TIDIER description [11]

	Audit and feedback	Educational outreach (supplemented by audit and feedback)	Computerised prompts and paper-based reminders
Materials and training	Practice-specific quarterly audit reports Each report contained a comparison of the practices’ behaviour or outcomes in relation to the other participating practices within their locality (i.e. their Clinical Commissioning Group responsible for commissioning services) and all participating practices across West Yorkshire to reflect on progress and to prompt the need for change. Information on clinical recommendations and potential change strategies were provided. Consequences of inaction were described. Practices were encouraged to set goals based on graded tasks (based on the number of clinical recommendations and number of patients to be targeted within each recommendation) and use an action planning template to detail who would do what; in what circumstances; and how and when the achievement would be reviewed. Subsequent reports included potential actions identified during outreach sessions. Computerised searches Clinical Information System (CIS) searches were available to systematically identify all patients whose care should be reviewed and facilitate repeat searching. Short and longer significant event audit (SEA) templates Short and longer forms were developed for risky prescribing and anticoagulation for AF indicators to facilitate root cause analyses and action planning from harmful events or near misses.	We commissioned for and recruited experienced Pharmacist facilitators who received 2 days training. Outreach sessions aimed to increase motivation, prompt individual and group reflection, increase confidence and intention to act. For each outreach visit, a practice-specific outreach pack was developed containing: the most recent (and all previous) audit report(s); a session outline; an action plan template that included space for noting current performance, setting a target, identifying who will do what and review date; and templates for assessing costs and benefits.	For risky prescribing nine computerised prompts were developed to be triggered within the consultation and during repeat prescribing on the basis of a clinical code algorithm for age/diagnosis/drug and duration. When triggered a brief message notified that the patient was at risk and presented one sentence of evidence-based risk (e.g. “This patient has CKD. NSAID use accounts for an estimated 15% of all cases of acute renal failure and 36% of drug-induced cases”). A one-click justification was required (e.g. continue with risk, add medication, or stop medication). Two prompts were developed for anticoagulation for AF but could not be made available within the study timelines. Patient-directed checklists Paper-based reminders in the form of laminated information sheets were created to convey key clinical information (blood pressure, risky prescribing and anticoagulation for AF). Pens and post-it notes were sent to all practices with a topic specific reminder to prompt behaviour.
Supportive activities	None.	Pharmacist training included a one-day face-to-face meeting with intervention developers focussing on goal setting, action planning, clinical barriers, and persuasive communication. This was followed by a half day of independent study using a folder of supporting documentation relevant to each clinical priority. The first outreach meeting of each facilitator was observed by an experienced facilitator and feedback was given.	None.
Intervention provider	Reports, searches and templates were created by the research team.	Professional outreach education company.	Reminders were created.
Mode of delivery	Reports were sent by post and e-mail. Practices were sent invitations to use computerised searches from a task from within their clinical information system. An email was sent from the ASPIRE team to the practice manager and colleagues introducing SEA templates.	Face-to-face sessions were offered to practices.	Practices were sent invitations to use computerised prompts from a task within their clinical information system. An email was also sent from the ASPIRE team to the practice manager and colleagues alerting them to option to accept the prompts into their CIS.
Schedule and intensity	Quarterly feedback reports. Practices were offered access to searches and SEA templates at the beginning of the study and reminded of their availability via quarterly feedback reports.	Practices were offered an initial 30-min session from April 2016. All practice staff involved in identifying/reviewing appropriate patients were invited to attend. A key clinical contact was identified to support practice engagement. Initial visits focussed on practice achievement data (from audit reports), identifying models of good practice, addressing barriers to change and creating an action plan to facilitate and review the change. Two days of pharmacist provision was offered to support patient identification and review. An additional follow-up visit was offered from 6 months to review action plan progress and support the practice to create more challenging or attainable plans.	Practices were offered access to prompts at the beginning of the study and reminded of their availability via quarterly feedback reports. Practices were offered access to checklists at the beginning of the study and reminded of their availability via quarterly feedback reports. Post-it notes and pens were sent to all practices.
Tailoring	Searches could be tailored by practices, allowing them to identify patients relevant to all or individual recommendations, or adjust target values to select specific groups of patients.	Session content could be modified to practice requirements.	Prompts could be copied and modified to practice requirements.
Modifications	None.		

To test this implementation package, we conducted two parallel, cluster-randomised trials using balanced incomplete block designs. Randomly assigned general practices received an implementation package targeting either diabetes control or risky prescribing in Trial 1 or targeting blood pressure control or anticoagulation in atrial fibrillation in Trial 2. Every practice was allocated to an active intervention, to balance any nonspecific effects across trial arms and thereby increase confidence that any difference in outcomes was attributable to the intervention [12].. We observed a significant, cost-effective reduction in risky prescribing and insufficient evidence of effect for the other three indicators.

### Process evaluation aim and rationale

Theory-based process evaluations of implementation interventions can identify factors that influence implementation and achievement of desired outcomes. We incorporated a parallel process evaluation into the trials to explore how real-life implementation compared with planned, theorised implementation. To do this, we collected fidelity and process data throughout and after the trial. We also chose one sociological theory (Normalisation Process Theory (NPT)) and one behavioural framework (TDF) that offered complementary insights into individual and group behaviours that influence implementation. Whilst the TDF [10] identifies the cognitive, affective, social and environmental determinants most relevant to implementation, its strength is identifying self-reported influences on capability, opportunity and motivation [13]. NPT [14, 15] provides an understanding of the dynamic social processes involved in implementation [16, 17]. NPT proposes that achievement is more likely when participants value the intervention (*coherence*), commit to engage (*cognitive participation*), commit staff and resources and work towards change (*collective action*), and appraise the package as useful (*reflexive monitoring*).

We sought to identify the social processes around implementation within primary care guided by the NPT; and the influence on hypothesised determinants (TDF) namely: *knowledge, beliefs about consequences, memory, social and professional role;* and *environmental context and resources* [8].

## Methods

### Study design and participants

**Fig. 1:**
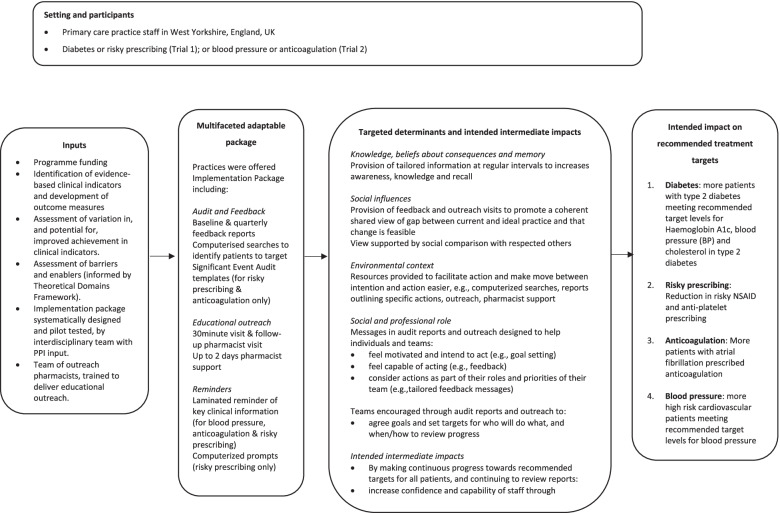
Multifaceted adaptable implementation package as planned

We used a multi-method approach comprising a longitudinal qualitative evaluation, a survey and an analysis of trial process data. Alongside opt-out trial recruitment, we recruited an additional eight practices from West Yorkshire, UK, to the qualitative evaluation. All were ineligible for the trials due to prior involvement in intervention development [8]. Process evaluation practices varied in list size and represented the geographical variation of the trial. Pre-intervention achievement was broadly comparable between trial and process evaluation practices across trials and indicators, with any variations reflecting the smaller sample of process evaluation practices [18]. Each practice was assigned a pseudonym and an independent statistician randomly assigned two practices to each indicator, balancing allocation by locality and practice list size (Table 3). Trial and process evaluation practices received the implementation package concurrently (Fig. 1).

**Table 3:** Process evaluation practices

**Practice**	**Identifier**	**Indicator**	**Geographical area**	**Approximate list size**
1	River	diabetes	Village	9000
2	Dale	diabetes	City suburb	10,000
3	Lake	blood pressure	Town	10,000
4	Hill	blood pressure	City suburb	5500
5	Valley	anticoagulation	Town	8500
6	Flower	anticoagulation	City suburb	15,000
7	Treetop	risky prescribing	City suburb	4500
8	Brook	risky prescribing	Inner city	23,500

A social scientist researcher (CH) independent of the trial team conducted the qualitative field work. She observed and collected data on how participants engaged with and understood the package, aiming to act as a non-participant observer. All trial data were analysed by an independent team of statisticians.

### Data collection

To assess fidelity, we collected data on delivery (extent delivered as intended), receipt (extent understood and engaged) and enactment (extent applied in clinical practice) of intervention components from trial and process evaluation practices (see Table 4 for summary) [19]. Fidelity was also tracked electronically (for e-mailed feedback and computerised searches), using structured logs kept by outreach facilitators and in process evaluation practices, via observational notes kept by CH. To assess fidelity further, in particular, to evaluate the visibility and enactment of intervention components in trial practices, we also surveyed all practices by e-mail after data collection. This post-trial survey was added to the study protocol as a further source of fidelity data when the trial was underway, and explored whether individual intervention components were received by practice staff, perceived as relevant, shared and discussed, and changed organisation of care. CH also held de-briefing conversations with outreach facilitators to explore their perceptions of intervention delivery and uptake.

**Table 4:** Fidelity of delivery, receipt and enactment for each intervention component

Intervention component and delivery mechanism	Sources of fidelity data	Receipt and enactment summary
**Audit and Feedback**		
Audit and Feedback reports Delivery: reports were sent by post and email every 3 months and taken to outreach meetings Computerised searches Delivery: organisation group on SystmOne - Practices were sent an email inviting them to join the organisation group to access the searches at any desired time. Alternately used as part of additional support from outreach facilitators Significant Event Audit Forms (for anticoagulation and risky prescribing practices only) Delivery: post and email with reports, and at outreach visits	**All practices:** Emails were tagged with “delivery” and “read” acknowledgement requests Fidelity survey asked staff to indicate if they received and read forms, and discussed them in their teams Outreach facilitators checked whether audit and feedback forms had been received (and recorded on structured logs) **Process evaluation practices only:** Researcher noted receipt and awareness of audit and feedback forms, observing where they were seen in practices and exploring practice staff views on the reports in one to one conversation and post-trial group feedback **All practices:** Computer system tracked whether or not the practices joined the organisational group and downloaded the searches. Outreach facilitators logged use of searches as part of additional support. Fidelity survey asked practices whether they were aware of and used the searches **Process evaluation practices:** researcher observed use of searches and spoke to practices in post-trial group feedback about the usefulness of searches **All practices:** Delivery tracked when delivered with audit reports or outreach visits (by outreach facilitators) **Process evaluation practices only:** researcher observed practices and asked practice staff about awareness and engagement with significant event audit forms.	All practices received reports, as tracked by email delivery and fidelity survey data Process evaluation noted variation in how reports were shared and used within practices (e.g. practice managers not sharing reports widely; only some practices discussing reports at meeting). 126 practices (87.5%) joined the organisational group and therefore could access searches. In the fidelity survey, 75% of trial and process evaluation practices stated they had used the searches. Receipt not specifically tracked in the trial practices, beyond delivery of reports and outreach visit. Searches were made use of in some process evaluation practices (for risky prescribing and anti-coagulation; infrequently for blood pressure and diabetes) In the relevant four process evaluation practices, there was evidence of receipt in one practice but no evidence of receipt or use in the others
Educational outreach meetings and additional support Delivery: personal visit to practice by outreach facilitator; offer made by phone and on each feedback report Maximum of two educational outreach visits were offered to each practice	**All practices:** Outreach facilitators completed structured logs recording who attended the training and their job roles. They also recorded which practices took up offer of additional support. Fidelity survey asked practices if they took up the offer of outreach support. **Process evaluation practices only:** Researcher noted receipt and awareness of outreach support, and asked staff about their engagement and the value of outreach meetings and support in one to one conversations and post-trial group feedback	Sixty-seven (47%) trial practices and seven (87.5%) of process evaluation practices received one outreach meeting. **Reasons given for not taking up outreach offer:** Trial practices declined because they were not interested, too busy or felt it was not needed. One anticoagulation process evaluation practice declined because they felt confident to do the work without outreach meeting. Additional support was taken up by 16 (24%) trial practices and five process evaluation practices. Most support was delivered remotely in the form of running searches, reviewing patient notes, and creating recommendations for management. Awareness of additional support was low in process evaluation practices (usually 1-2 staff members being aware of it). Eight (5.6%) trial practices and three process evaluation practices received a second visit, another requested a visit but this could not be accommodated before trial end. Significant delays noted in delivering outreach visits to practices.
**Reminders**		
Computerised prompts (available for risky prescribing only) Delivery: organisation group on SystmOne - Practices were sent an email inviting them to join the organisation group to access the prompts at any desired time.	**All practices: **Computer system tracked whether or not the practice downloaded the prompts Fidelity survey asked practices if they used the prompts **Process evaluation only:** researcher asked staff about their awareness and engagement with the prompts	Eight (32%) trial practices and both process evaluation practices downloaded the risky prescribing protocol Evidence from process evaluation practices that the prompts were considered useful by one practice as they enabled greater involvement of staff typically not involved in risky prescribing decisions.

For the qualitative evaluation, CH met with practice staff prior to intervention delivery to establish rapport and a sense of pre-intervention context. She collected observational (e.g. practice meetings), documentary (e.g. clinical protocols, letter templates etc.), and interview data related to awareness and use of the implementation package over 12 months at each practice. NPT and TDF constructs informed fieldwork, guiding but not delimiting data collection [20].

CH conducted individual semi-structured interviews with the relevant clinical lead, practice manager and other staff involved in the organisation or delivery of care for each indicator at two time-points in each practice. Initial interviews explored roles and responsibilities, barriers to achievement and early responses to the implementation package (Appendix 1. Longitudinal interview guide). Follow-up interviews throughout the intervention period explored the perceived usefulness of the package over time. All interviews were audio-recorded and transcribed verbatim.

CH used field notes to record informal conversations with staff, observations in non-clinical areas, of relevant practice meetings, and outreach meetings (Appendix 2. Observational guide).

Practices were prompted to collate indicator-related documents (e.g. treatment protocols, letter templates, patient leaflets and minutes from practice meetings) in a study box given to practice managers. Practices chose which documents to share with the researcher; related documents were reviewed at the end of the study.

CH conducted focus groups with each practice towards the end of the study to reflect on their overall experience, intended indicator work, and what did and did not support implementation (Appendix 3 Interview guide for final practice meeting). Practice managers were asked to invite relevant staff.

### Data management and analysis

Interview transcripts and detailed field notes were anonymised and managed in *NVivo 10* (QSR International, Warrington, UK). We developed a coding framework (Appendix 4 Table 9 Normalisation Process Theory (NPT) coding dictionary and Appendix 4 Table 10 Theoretical Domains Framework (TDF) coding dictionary) with inductive and deductive elements guided by NPT and TDF constructs [21, 22]. We created chronological practice narratives and process models for each practice after an initial directed content analysis. The narratives outlined delivery, exposure, and enactment within each practice over time and the process models illustrated the implementation processes within practices and their interactions with the components. CH undertook coding and constructed the practice narratives and process models. These were reviewed and refined iteratively in multi-disciplinary research team meetings (with experience in social sciences, implementation science and primary care). To explore fidelity, we compared practice process models with an idealised process model which outlined implementation as intended to identify and theorise delays and unintended consequences of the intervention.

Descriptive quantitative fidelity data collected from all trial and process evaluation practices informed interpretation of the process evaluation practice narratives. For the post-intervention survey fidelity was considered high if practices received feedback reports, accepted outreach, and accessed computerised searches; medium if they received feedback and either accepted outreach or accessed searches; and low if they only received feedback reports.

**Fig. 2:**
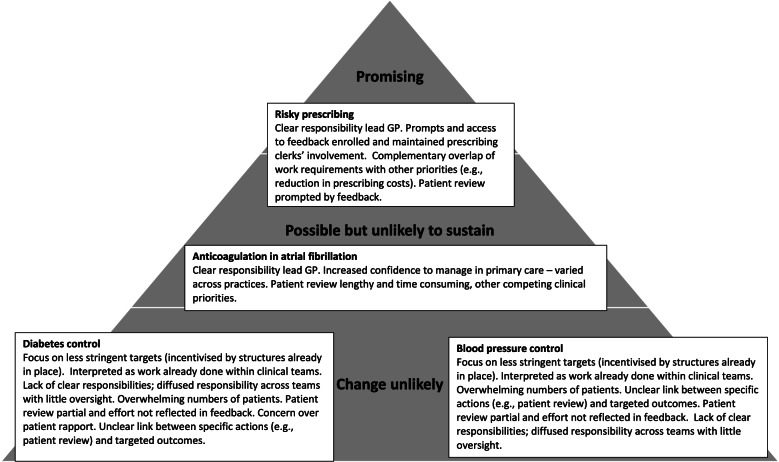
Comparing and contrasting engagement and predicted achievement in the four packages (categories predicted prior to trial results and confirmed by trial findings)

We conducted analyses and constructed practice narratives before the trials analysis in February 2017. We then interrogated the practice narratives further in the light of the trials findings in June 2017 (Fig. 2).

## Results

We collected data from 144 trial and eight qualitative evaluation practices. CH conducted 64 interviews with practice staff, approximately ten hours of observation, and an end-of-study focus group at each process evaluation practice. Fifty-nine staff from 57 practices (38% of all trial and process evaluation practices) responded to the post-trial fidelity survey. CH interviewed all 15 outreach facilitators.

We prospectively identified three patterns of intervention exposure and enactment which help explain the success in reducing risky prescribing and the failures to improve the other three indicators. These patterns were that (i) the intervention achieved integration by meeting the needs of the practice and sustaining collective action, (ii) exposure to the intervention limited engagement and pace of new ways of working and (iii) there were drops or delays in action as unintended consequences of intervention components and their delivery. We illustrate these three patterns with examples from practices, drawing on NPT and TDF to situate these patterns theoretically. Figure 2 presents an overview.

Pattern 1 Achieving integration: meeting the needs of the practice and sustaining collective action (Table 5).

Staff in practices that targeted risky prescribing and anticoagulation considered that the intervention package supported change in important clinical areas; it both aligned well with practice goals and was sufficiently differentiated from what practice staff were already doing. Staff found the feedback reports informative in both showing how their achievement compared with that of other practices and highlighting the consequences of change. The feedback also appeared to leverage social influence effectively.

The Chair said ‘yes, I think it made it much clearer what the risk is, that you were actually saving people’s lives by anticoagulating’

### Observation, final practice meeting, Flower (anticoagulation)

[GP partner] commented on the quote on the side, mentioning that [the expert quoted] was a known atrial fibrillation expert—knows what he is talking about

### Observation, Valley (anticoagulation)

Outreach was a critical time for enrolling staff in the intervention. Facilitators generally perceived outreach sessions for risky prescribing and anticoagulation to be successful as participants were involved in relevant clinical work. This was perhaps easier to achieve for risky prescribing and anticoagulation, in comparison to diabetes and blood pressure. Practices identified fewer staff members (e.g. clinical lead prescriber or in-house pharmacist) with clear divisions of labour as critical to the organisation and delivery of care.

In contrast to diabetes and blood pressure practices, risky prescribing and anticoagulation practices had comparatively few patients to review (approximately 200-300 vs approximately 30-50, respectively). This meant that risky prescribing practices did not require substantial re-organisation of resources or working patterns to review patients. Anticoagulation staff began to re-organise resources to review patients previously reviewed in secondary care. Our computerised searches facilitated this, providing staff access to patient lists.

Only risky prescribing practices were observed re-directing staff resources into regular computerised searches. Prescribing clerks in one such practice started alerting doctors to review repeat prescriptions following computerised prompts; the other practice disabled the prompts during consultations as they were considered disruptive.

One risky prescribing practice implemented repeat audits, which may have enabled a continuous feedback loop and helped sustain the work. In this practice, it was notable that the searches were considered useful and perceived as routine work for the prescribing clerk and practice manager. Moreover, there was evidence that each staff member’s role was clearly outlined from the first intervention-related meeting and staff trusted each other’s capacity and ability to engage with this work over time.

We did, we searched once every month (...) And then we reviewed, brought in all those patients in that we hadn’t…treated that were on the recall list that we hadn’t treated, and we reviewed them. So, although it’s more work (...) We were on top of it

### GP lead, interview, Treetop (risky prescribing)

The intervention package seemed to meet a perceived need in risky prescribing and anticoagulation practices, providing desired information about the topic, trusted evidence of the consequences of action, and motivation to change practice. In addition, staff collectively believed they had the capability to achieve what was required. It was relatively easy for practices to identify key people to carry out the work, and substantial re-organisation of resources was not required. For risky prescribing practices in particular, it seemed that the searches and (to a lesser extent) prompts made collective action both more feasible and sustainable.

**Table 5:** Achieving integration and collective action: TDF and NPT in practice

The feedback reports enabled change by targeting gaps in knowledge around risky prescribing and anticoagulation (TDF *knowledge*); data illustrating the importance of the topic stimulated a belief that the work was valuable (TDF *beliefs about consequences*); the small numbers needed to treat enabled a sense of the work as achievable (TDF *beliefs about capabilities* and *environmental context and resources*). The intervention met a perceived need and outlined clear individual and communal expectations (NPT *communal and individual specification*) without replicating current practices (NPT *differentiation*). The anticoagulation reports traded on appropriate expertise to encourage practices (TDF *social influence*); in risky prescribing, comparison with other practices stimulated competitiveness (TDF *social influence*). Those involved attending outreach meetings and reviewing reports were appropriate in terms of clear roles and skills to do the work (TDF *social and professional roles* and *skills*). This enabled staff to outline work needed quickly and efficiently, targeted at the right people (NPT *initiation*, *enrolment*, and *legitimation*). Staff worked together and had clear ideas of who did what (TDF *social and professional roles*; NPT *interactional* and *skill-set workability*). Risky prescribing practices that repeated the searches saw the impact of their work (NPT *systematisation*). As the trial went on, positive feedback on achievement encouraged continued engagement (TDF *motivation* and *emotions*). Discussing this feedback enabled continued work (NPT *activation*), as well as reinforced a sense of how feasible and useful the work was in practice (NPT *individual* and *communal appraisal*).	

Pattern 2 Limited coherence: not targeting the right determinants and outcomes (Table 6).

The diabetes and blood pressure practices were initially enthusiastic about the intervention and were observed discussing reports in practice meetings and participating in outreach sessions. These practices had clinical leads and numerous clinical and administrative staff involved in care around these indicators. However, it soon became clear that the intervention tended not to fit with practice teams’ perspectives and needs in two significant ways.

First, practice staff felt they were already aware of, and working towards, achievement in these areas. The collective view tended to be that the practices had invested significant resources into delivering care in these areas and that there was no capacity—and little incentive—to change existing structures. Practices tended to believe that they already knew what was needed, there was little value to be gained in changing their systems and processes, and the intervention components did not add value to their work. The intervention was therefore not experienced as bringing anything new to the practices, and little effort was expended in considering change to work organisation within the practice team.

[GP partner said] we have got good systems, patients do get reviewed (…) we need to make sure [blood pressure] doesn’t break our systems.

### Observation, educational outreach, Lake (blood pressure)

Second, practice teams drew on discourses around the feasibility and desirability of achieving the targeted outcomes. Outcomes targeted in the study for diabetes and blood pressure involved a composite endpoint (HbA1c, blood pressure and cholesterol) and achievement of recommended blood pressure levels in patients at high risk of cardiovascular events, respectively. These outcomes tended to be more ambitious than those required for the Quality and Outcomes Framework (QOF), an existing incentive scheme for UK primary care [23]. Within the practice teams, there were evident splits, with some staff seeing these more ambitious targets as desirable and pushing for additional work to meet them, and other staff considering themselves at capacity and stricter targets potentially damaging to patient rapport. Intervention-related discussions raised the policy context (where practices are remunerated for meeting QOF targets and perceived as under-funded) as well as current team contexts (in terms of skills, capabilities, and roles). Although there were champions of the intervention in some practices, there was little evidence of a shared coherent vision of its value or of clear agreed changes to staff roles and responsibilities or sequencing of interdependent team-based behaviours.

at the outreach meeting, the practice had discussed adding in some hypertension work during the flu work and he said yes, he remembered, but that was wildly unrealistic

### Observation, GP interview, Hill (blood Pressure)

[It] was targeting too many patients, they didn’t have the resources. The chair agreed, when you see a list to review of about 100 patients, your heart sinks. The PM said we refined the searches, then the pharmacist looked at it, and then about 30 people were on the list given to the diabetes lead so he could look if it was clinically worthwhile to doing anything with them. He said that they just don’t have the time or capacity

### Observation, final practice meeting, River (diabetes)

Whilst the majority (143; 94%) of trial and process evaluation practices created an action plan during outreach, facilitators reported that practice staff varied in their ability to select targets and set manageable goals for indicators which included significant numbers of patients. Action planning seemed more challenging for diabetes and blood pressure practices as the workload extended across the whole practice and patient lists were large. In particular, diabetes and blood pressure outreach sessions were often delivered during routine practice meetings and were challenging to manage due to the large number of attendees, each with greater or lesser incentive and role in completing work regarding the indicator. Action plans resulting from these sessions tended to be considered less feasible by facilitators in that they rarely specified named individuals for specific work or allocated a date for reviewing progress.

**Fig. 3:**
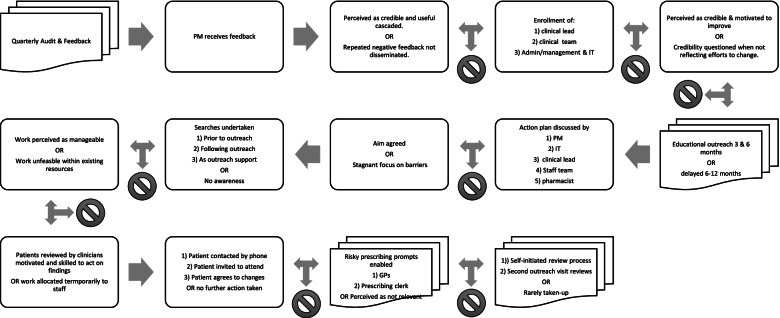
Fidelity of delivery and engagement as intended and observed variations indicated by stop signs

Ultimately, most staff in these practices only engaged passively with the intervention continuing to work to established targets and structures (see Fig. 3). There was little evidence that searches or outreach support contributed to changes in the organisation and engagement. Where there was engagement, it typically was not organised in a sustainable manner (e.g. one practice used a medical student as an extra resource rather than assign a role to a permanent staff member).

The GPs said they discussed the reports, it possibly raised the consciousness, but that’s it

### Observation, final practice meeting, River (diabetes)

It worked really well while I had my student over the summer (…) I think we made a massive improvement at the beginning and then it’s sort of tapered off as [we] just couldn’t keep the momentum going I think

### Lead GP, interview, Lake (blood pressure)

**Table 6:** Failure to Cohere: TDF and NPT in practice

It was felt that the practices knew about the topics and already worked hard to achieve outcomes (TDF *knowledge* and *beliefs about capabilities*). It was felt little more could be achieved (TDF *beliefs about consequences*, *motivation* & *emotion*). The intervention did not seem to add value or seem different to the practice’s existing work (NPT *differentiation*) and staff felt it was more or less important to work to the specified intervention targets (NPT *communal and individual specification*). Practices felt they did not have the resources or the incentive to change systems and processes (TDF *environmental context and resources* and *motivation;* NPT *coherence*). Outreach meetings tended to involve large numbers of staff with varied interests and desire to engage in work (TDF *social and professional roles* and *skills*). Levels of participation in work around the intervention varied (NPT *cognitive participation: initiation and enrolment*), and there was little sense of the intervention being integrated into staff routines or influencing the allocation of resources (NPT *collective action: relational* and *contextual integration*) due to how staff perceived the intervention as irrelevant to their role (TDF *social and professional roles*) and unlikely to impact on patient outcomes without negatively affecting patient rapport (TDF *beliefs about consequences*).	

Pattern 3 Drops or delays in action: unintended consequences of the intervention components and their delivery

Across all indicators, we observed how specific intervention components were delivered and received created unintended consequences that impeded implementation. Delays and difficulties in delivering outreach and associated pharmacist support impeded the ability of practices to improve their achievement. Figure 3 provides an overview of observed variations.

Outreach was perceived as important and frequently viewed by practices as the intervention starts. This was an unintended consequence of the outreach offer, as outreach was conceptualised by the intervention team as an adjunct to audit and feedback (delivered at the start of the intervention) rather than the main intervention component. To complicate matters further, it was intended that practices would receive an initial outreach meeting within the first six months of the intervention period with a follow-up visit between six and twelve months. Thirty-eight (57%) trial practices received a visit as intended (i.e., during months one to six) and 29 (43%) initial visits took place in months six to 12, limiting time available for implementing changes. Data from outreach facilitator logs suggested that delays were mostly due to ensuring key clinician availability and lack of meeting space, rather than availability of facilitators. Notably, many practices sought to ensure key clinicians were present, demonstrating their engagement with educational outreach as important. The combination of practices perceiving the outreach visit as the intervention start coupled with delays in delivery meant that practices enacted fewer changes than hoped for in the earlier months of the trial, which had an impact on potential for indicator improvement. In addition, facilitators did not have access to electronic health records to prepare for the outreach meeting, limiting their ability to discuss patient-specific barriers, facilitate goal setting and initiate action. For some practices, the delay in accessing outreach meant they did not actively engage with the intervention until over halfway through the intervention year.

we’ve kind of waited for [outreach support] to happen (…) I hadn’t appreciated that we actually needed to be chasing that up and organising it!

### Lead GP, interview, Dale (diabetes)

Only sixteen (24%) of the trial and process evaluation practices that received an outreach visit were offered two days of pharmacist time to enable patient identification and clinical review. This support was mostly delivered remotely by a dedicated pharmacist, not by the visiting facilitator as planned. Moreover, outreach support could not be delivered within the first six months as intended; consequently, this delayed action in those practices that waited for assistance, and then limited the time available for actions to be implemented and take effect. This may be particularly relevant to diabetes and blood pressure practices that felt unable or were resistant to act due to greater patient numbers.

Reminders (of blood pressure targets, risky prescribing and anticoagulation contraindications) were rarely observed in the qualitative evaluation practices and seldom recalled by staff.

The diabetes and blood pressure practices mostly did not identify a need to change their work organisation. Diabetes and blood pressure trial outcomes were perceived as ambitious as they were based on achievement of a composite set of indicators. Composite indicators identified larger patient numbers for review for already stretched staff. However, they did continue to review the reports, comparing their achievements to other practices on the targeted outcomes. This repeated feedback had the unintended consequence of generating negative emotion in some practices, and ultimately de-motivating staff who then disengaged from the intervention or questioned the value of changing practice.

He said that they had felt like they had done quite a lot of work but this was not reflected in the figures. He laughed as he said it, it felt a bit dispiriting really. He felt they were doing so much work just to stay in the same place. Other people nodded and agreed

### Observation, final practice meeting, River (diabetes)

In one practice, the practice manager stopped disseminating reports when there was no significant positive change in achievement. The intervention therefore became less visible within the practice over time.

I mean to be honest with you normally we’d share it with all the partners, but because the results didn’t look that good to me, I didn’t want to embarrass [GP - diabetes lead] by giving it to all the partners

### Interview, Practice manager, Dale (diabetes)

Not only did the intervention fail to target the right determinants and fail to differentiate itself from routine work, it also stimulated a negative emotional response as it gave practices feedback that did not reflect their perceived efforts around those indicators. This also meant that the intervention lost its influence over time, as the staff either actively avoided the data or questioned its value or accuracy.

**Table 7:** Unintended consequences: TDF and NPT in practice

For diabetes and blood pressure practices, the intervention failed to differentiate itself from routine work (NPT *differentiation*), and practice staff perceived themselves as already doing this work (TDF *social and professional roles)*, lacking resources or capacity to do any more work (TDF *environmental context and resources*, *beliefs about capabilities),* and unlikely to achieve anything more by engaging with the intervention (NPT *communal specification*; TDF *beliefs about consequences*). Some staff perceived its value (NPT *individual specification*) but were unable to gain traction with other team members (NPT *cognitive participation: enrolment* and *legitimation*). Delays in delivery of outreach and outreach support had the unintended consequence of delaying practice participation and access to trial resources (TDF *social influences* and *environmental context and resources*), reducing the likelihood that staff would have time to adopt changes in their work (NPT *collective action*: *contextual integration*) or enrol in the work (NPT *cognitive participation: enrolment*). Feedback reports had the unintended consequence of de-motivating staff as they failed to achieve change on the more ambitious indicators (TDF *motivation* and *emotion*) and staff reacted by reducing visibility of the intervention (TDF *memory*) or believing the intervention to be ineffective or not worth engaging in (TDF *beliefs about consequences;* NPT *collective action*: *relational integration*).	

## Discussion

We observed three main patterns that may explain why an adaptable implementation package was effective in improving care for one out of four targeted indicators. First, in *integration and achievement*, the package “worked” when it was considered distinctive and feasible. Timely feedback directed at specific behaviours enabled continuous goal setting, action and review, which reinforced motivation and collective action. For one indicator (risky prescribing), the social processes and behavioural determinants matched well and had the desired impact of increasing motivation and action in the desired direction. Second, *impacts on team-based determinants* were limited, particularly when the complexity of clinical actions impeded progress*.* In these cases, the intervention targeted an area of agreed clinical need but was not adequately tailored to the complexities of team dynamics and systems. Third, there were *delivery delays and unintended consequences*. Delays in scheduling outreach and an unintended overemphasis on the status of outreach reduced ownership and time for improvement. As a consequence of delayed action, receiving repeated stagnant or declining feedback also undermined engagement.

A recent mixed-method process evaluation suggested that the combined use of psychological and sociological theory increased the explanatory potential of a hospital-based process evaluation [24]. One novel feature of this study is that we compared general practice responses for four different evidence-based indicators targeted by an adapted implementation package with common components and behaviour change techniques. Our findings suggest the importance of selecting indicators that have clear actions for individuals. Complex indicators involving a sequence of interdependent team behaviours to change systems of care were less successful. Our use of both frameworks allowed us to create richer explanations of behaviour at both group and individual levels and how these levels interact, which was valuable for our comprehension of the trial outcomes. We illustrate how under particular conditions, the implementation package achieved integration and collective action, failed to cohere, and led to unintended consequences (Tables 5, 6, and 7). Using the TDF constructs allowed us to specify the relevant implementation behaviours to attend to in context whilst NPT generated an understanding of the process dynamics, both of which are required to inform the specificity for designing future implementation strategies.

### Study limitations

Given the challenges of prospectively identifying patients consulting for four different indicators, no patient consultations were observed. Instead, we focussed on the perspectives of those directly involved in delivering care. We chose not to collect questionnaire TDF and NPT data (given the challenges of operationalising these items for a complex multi-component package) from the wider practice team, instead focussing on the perspectives of those directly involved. As the trials progressed, we noticed in the process evaluation that there were gaps in our knowledge of intervention component receipt and enactment. Structured logs captured awareness of audit reports at outreach support visits but awareness and use of other components were more difficult to track due to the autonomy of practices to access at any time; we added a post-trial fidelity survey to explore this more specifically across the trial practices.

TDF alone was used in the development of the implementation package, identifying common determinants from interview data. Our understanding of group processes and how determinants might interact was likely limited by this approach.

### Implications for practice and research

We suggest several lessons for the design, delivery and evaluation of implementation strategies based on our findings (Table 8).

**Table 8:** Where should intervention designers and evaluators direct their efforts and resources?

Stage	Lesson
Selecting indicators	Consider fit with professional values, patient benefit and practice goals to enable a clear understanding of the need for something to be done differently and that improvement is possible Consider workload of reviewing patients near to targets (e.g. impact of stringent targets on patient preferences and rapport) and how this fits with achievement Ensure outcome measures are sensitive to efforts to improve achievement to enable learning from working to achieve change Limit the number of indicators and specify clear corrective actions or behaviours that will have impact on achievement Make visible individual contributions towards changing team-based behaviours and enable individuals to be accountable to themselves and their team
When developing intervention components	
Audit and feedback	Identify a named lead to coordinate the overall plan and individual actions Facilitate reach to those who are able to act to improve performance and suggest that feedback is made visible in the practice and at practice meetings Make clear relevance to non-clinicians Focus on feedback for learning in addition to feedback on performance (i.e. what could be done differently in addition to feedback on gap between actual and desired performance to support underachievers) Frame behaviour to showcase benefit of additional or modified ways of working (e.g. reduce unwanted actions (e.g. reduce risky prescribing or reduce strokes) as opposed to increase desired behaviours (e.g. increase prescription of anticoagulation)) Action plans that suggest specific and feasible actions could minimise cognitive load and overcome habitual patterns of working Consider reporting timeframe in relation to work to be undertaken. Estimate timeframes required for actions on action plans and time feedback accordingly Repeated negative feedback may be dispiriting and decrease ownership
Educational outreach	Provide a time to review audit feedback and conduct patient-identifiable searches before meeting face-to-face to further explore barriers and goal setting Enrol all potentially relevant staff (e.g. administrative, managerial and clinical) as early as possible to create a sense of ownership and maximise time for improvement Create an open discussion of problems, how individuals work and ways to overcome challenges Ensure that the facilitator is seen as credible
Reminders	Patient identifiable searches may reduce burden and enable practices to develop a continuous feedback loops to track and maintain improvements Ensure that searches and computerised prompts can be easily adapted to focus on practice targets for achievement Computerised prompts may be applicable to both clinical and administrative staff involved in repeat prescribing
When delivering interventions	Establish commitment, rapport and mobilise resources prior to intervention delivery (e.g. time commitment, access to identifiable audit data) to increase awareness of intervention package Identify a practice lead who can empower participation and manage competing priorities Establish a team including management, clinicians and administrators to reinforce collective action Encourage rapid actions in intermediate process and outcomes to make progress visible and increase internal motivation to continuously improve Consider opportunities for social exchange of success stories of what others are doing
When evaluating implementation components	Enable interactive communication between intervention developers and practices to support tailoring and adaptation of interventions to context Pilot test delivery, receipt and engagement as informed by NPT and TDF constructs before evaluating at scale

*When selecting or developing indicators of achievement* consider their fit with professional values, patient benefit and practice goals to augment motivation to change. Limiting the number of indicators and associated corrective actions needed to be undertaken by different actors may support collective action. Framing indicators to showcase the benefit(s) of additional or modified ways of working (e.g. reduce unwanted outcomes such as strokes) as opposed to increased work (e.g. additional consultations and prescriptions) may enhance motivation. Indicators that specify clear corrective actions which are sensitive to efforts to improve, may enable rapid learning from changes. Whilst we sought to augment work already undertaken, this resulted in unintended consequences (e.g. impact of stringent targets on patient preferences and relationships) of reviewing patients near to targets.

*When developing intervention components*, it may help to clearly differentiate the additional work required by the intervention from pre-existing work. Most practices were already engaging in alternative approaches to improve achievement and may be experiencing “intervention fatigue” [25], limiting capacity for enactment. Differentiation was enabled by the environmental context and resources of the practices as well as by staff beliefs about their knowledge, skill and capabilities. Where the links between specific staff actions and achievement were more direct and clearer, staff seemed motivated to act, clear about their roles and responsibilities, and more likely to stay engaged with the work over time. Practices requested social exchange of what others are doing to influence achievement.

It is important to consider how practices will perceive and value different intervention components in combination, and exploring this with think aloud interviews at pilot stage could be of benefit [26]. TDF was useful to identify the relevant determinants but could not predict the direction or size of their impact in context or combination. The trial underestimated the weight practices would place on face-to-face elements of the intervention (e.g. outreach visits); this could have been predicted through piloting the package to explore theoretical “fit” of an implementation package at both the individual and group level. Using sociological and psychological theory together in the piloting stage may have enabled some unintended consequences of the process of intervention delivery and group sense-making to be identified and planned for.

Process mapping all of the relevant behaviours required by staff and patients may support the design of a more cohesive package. Changing diabetes and blood pressure outcomes involved a longer interdependent chain of actions from disparate individuals to collectively review notes, recall patients, conduct patient consultations; and motivate patient behaviour change. Our package was not designed to engage patients.

*When developing feedback interventions* estimating the time staff need to receive and act on feedback can guide the timing of feedbac k[26]. Making patient identifiable searches easy to adapt could allow practices to focus on their targets for achievement, and enable continuous feedback loops to track and maintain improvements. Feedback that suggests specific and feasible actions could minimise cognitive load and overcome habitual patterns of working [26]. Making visible the individual contributions towards changing team-based behaviours within feedback could increase normative accountability. However, repeated negative feedback may be dispiriting, decrease credibility and restrict dissemination of subsequent feedback. Feedback developers could consider alternative methods of presenting negative or unchanging feedback data that reflects effort expended in all parts of the implementation chain (e.g. reviewing patient notes).

Educational outreach allows for further flexibility and individual tailoring in delivery. Conducting patient-identifiable searches prior to meeting face-to-face can facilitate an open discussion of problems, how individuals work, and ways to overcome challenges. It is important to ensure that the facilitator is seen as credible in these discussions. We tendered for a company with expertise in delivering primary care outreach. Our pragmatic trial illustrates the challenges in organising meetings with practice staff who have limited opportunities to engage with improvement work. Computerised prompts with accompanying guidance for tailoring to clinical and administrative staff may prevent prompt fatigue.

*When delivering intervention components* our analysis suggests that interventions were not necessarily received by the people who could enact change. Identifying and enrolling a practice lead to coordinate dissemination of multi-component interventions, with the opportunity to continuously review their impacts, may improve effectiveness [27]. Future researchers could review baseline data or engage with practice staff to identify delays in delivery or misconceptions about intervention functions. During intervention, development consider the “hidden” contributions of non-clinicians to uptake and enactment [28]. We suggest specifying the relevance of interventions to named non-clinicians and clinical leads to facilitate intervention reach to those able to improve achievement. We also suggest frontloading the delivery of components deemed most important by practices (as identified in piloting); outreach visits were more influential than intended and hardest to deliver, resulting in a negative impact on implementation.

*When evaluating implementation strategies*, decisions have to be made as to when and how to evaluate promising interventions. We suggest that formative process evaluations are vital to enable a full understanding of all direct and indirect risks and impacts associated with intervention delivery, reach and uptake prior to rigorous evaluation. Whilst we pilot tested intervention component acceptability, we did not examine whether the package could support practices to improve achievement. This study demonstrates the value of integrating psychological and sociological perspectives in a process evaluation, particularly the likely impact of an intervention on individual and team behaviour change, prior to evaluation. Intervention developers could use NPT and TDF in adaptive designs to rapidly collect sufficient data to understand if interventions should be evaluated, refined or abandoned in advance of definitive trials [29, 30].

## Conclusions

We drew upon the Theoretical Domains Framework and Normalisation Process Theory in a longitudinal study to explain the variable success of an adaptable implementation package promoting evidence-based practice in primary care. The package appeared to work best when it was distinct form and yet easily integrated within existing organisational routines, with clear direct patient-level benefits. It failed when delivery was delayed and professionals could not observe or did not expect any improvement resulting from their efforts.

## Data Availability

The datasets generated and/or analysed during the current study are not publicly available as the data may identify practice staff. Anonymised data may be made available from the corresponding author on reasonable request.

## Appendix

### Appendix 1 Longitudinal interview guide

**Initial interview**

Role and Context

What is your role in the practice? What are your main responsibilities?

Who do you tend to interact with most in the practice? (e.g. nurses, doctors, PM, reception, patients etc.)

How often do you tend to be here? (e.g. how many shifts/hours a week, any commitments outside the practice like CCG involvement)

Do you attend meetings within the practice or with other staff in the practice?

What involvement do you have in providing or organising care for [clinical topic]?

Barriers/Facilitators to best practice in specific clinical topic

Recently in the practice, have there been any changes around [relevant clinical topic]? What has motivated or influenced these changes? What impact have these changes had on you, your workload or on work in the practice?

In your opinion/experience, what do you find to be the barriers around implementing best practice in [clinical topic area]?

Explore any of the barriers in further detail, probe around meaning and for specific examples

In your opinion/experience, what enables changes to practice, especially around adopting best practice?

Can you think of an example where you implemented a successful change, relevant to the clinical topic? How did this come about? What do you think made it successful?

*Areas to probe around:*

*Capability (Skills, Knowledge, Psychological, Behavioural Regulation)*

*Motivation (Social role/identity, beliefs about capabilities, beliefs about consequences, motivation, emotion)*

*Opportunity (Social influences, environmental context/resources)*

Awareness of and expectations around ASPIRE

How aware of the ASPIRE research programme are you? Have you attended any meetings or had any conversations with others in the practice about ASPIRE?

[If aware of ASPIRE]

What do you know about ASPIRE?

What do you hope or expect from involvement with ASPIRE?

Does ASPIRE make sense to you? Are there any parts of ASPIRE that do not make sense or you feel could be improved?

[If unaware or has forgotten], ASPIRE aims to help practices provide care in line with best practice by using a number of quality improvement tools like audit and feedback, educational outreach and computerised searches and prompts

Who in the practice would know about this? Why do you think they would know about it? What is their role?

What experience do you have of [searches/prompts/audit and feedback/outreach]? Do you think they help to improve the care offered to patients in relation to [clinical topic]? [If yes], in what way/s? [If not], in what way/s have they not been useful? [Follow up by exploring barriers, q7]

Audit and feedback form/s

[Have copy or copies at interview] The practice was sent an audit and feedback form by email and post in [month] (and then on other dates…)

Have you seen the audit and feedback forms? [If yes] How did you end up seeing them? What did you think of it/them? Was there anything useful about it/them? How could it have been more useful to you? Did anything about it surprise you? Anything not make sense to you?

Was the audit and feedback form or forms, to your knowledge, discussed within the practice? If so, how was it discussed and who was involved in this? Were you involved?

[If no, show the form to the participant] What do you think of the form/s? What looks useful to you (and in what way/s is it useful)? Is there anything surprising or unclear in it?

Was there anything in the A&F forms that you decided not to work on in the practice? If not, why not?

Educational outreach

Your practice had an outreach session on [date], did you attend the meeting?

[If yes], what did you think of the meeting? What seemed useful to you? Was anything raised unexpected or new to you? What (if anything) stood out from this meeting?

[If no], are you aware of what happened at the meeting? Did anyone tell you about it? Do you think it was relevant to you? Why were you unable to attend?

What has happened after the meeting? Was an action plan agreed? Did you have any actions as a result of the outreach? Were any changes made to practice? Has this affected you in any way or affected any part of the practice’s work?

Other ASPIRE elements *(more relevant at 2*^*nd*^ *interview phase)*

[For risky prescribing], have you come across any computerised prompts for prescribing safely? What are the prompts like? How do they work? Are they useful to you (if so, how)?

[All practices], ASPIRE have provided searches to identify relevant patients—have you come across these? Are these useful (and in what way/s)? [If not], would you find this sort of thing useful?

[For hypertension/diabetes], have you seen a patient checklist from ASPIRE to be used in consultation with patients? What do you think of this type of tool? Is it helpful, and if so, how is it helpful?

[For prescribing/AF], have you seen ASPIRE significant event audit forms? Are you familiar with significant event audits? Do you find them helpful for improving patient care, and if so, how? Have you experienced any difficulties with significant event audit - any barriers to doing or using them?

[All practices], have you seen any pens, post it notes or laminates provided by ASPIRE? Do you feel these are useful? [If yes], in what ways? [If no], are these types of reminders ever useful? Can you think of any times when any of these things have been useful to you in the practice? [Probe for detail—why were they useful at that time, how did you use them, where did they come from etc]

[All practices], ASPIRE are offering some support from a pharmacist to help work on the clinical topic. Are you aware of your practice being offered or taking this offer up? What do you think of this offer?

[If not aware of offer] would you find this useful?

[If the support has been experienced], did you find this useful, and if so, how? What did the facilitator/pharmacist do? Which members of the team did they liaise/ work with?

Implementation

To your knowledge, has anything in the practice been done differently as a result of involvement in ASPIRE?

If yes, explore what has been done differently, who is involved in the changes and what the intentions are behind the changes

Are you aware of any plans to do anything differently in the practice as a result of ASPIRE?

If yes, explore what plans there are, who is involved in them and what the intentions are behind these plans

Has involvement in ASPIRE affected you or your work in any ways? If so, how? Has it had any impact in the practice in general or any impact on anyone else (that you are aware of)?

Drill into specific details

Close

Who else in the practice do you think would be good to talk to?

**Second interview**

*Change over time*

How have things been in the practice since we last spoke/since I was last in the practice?

Have there been any changes in the practice recently?

any staffing changes

any changes in what you do in your role

any changes at an organisational level

any new initiatives or research taking place in the practice (e.g. initiatives in the CCG and local area)

(If there have been any changes) In what ways have these recent changes affected the practice? Have they affected your role? Have they affected the delivery of care? Have they affected the atmosphere in the practice? How?

*Clinical topic*

How is care organised around [diabetes/hypertension/prescribing NSAIDs, atrial fibrillation]? Who is involved in this?

Are you aware of any changes in the practice around care for this topic or provision of services for this topic? Have there been any changes to your role in relation to this topic?

If there have been changes, what has motivated these changes? How have the changes affected care, your role, other practice staff, the patients?

What effect (if any) do you think these changes have had on the practice’s achievement for that clinical topic? Have the changes affected anything else, e.g. care in another area?

*Intervention components*

How aware have you been of ASPIRE in the last couple of months? Have you seen or used any of these components?

Reports—paper/email *(take examples)*

ASPIRE searches

Prompts (if relevant)

Any emails or visits from ASPIRE (esp. outreach support, outreach visits, CQC/QOF communications)

Pens, post-it notes, laminates (if applicable)

SEAs (if applicable)

Anything else relating to ASPIRE (e.g. ASPIRE box)?

If not aware/involved, did you feel that this was important/relevant to you?

(About different components) In what ways were these used? Who has used them? Were they discussed formally or informally? Have they had an impact on your work in any way, or on anyone else’s work in the practice?

(If they had educational outreach or drew up an action plan) Are you aware of any activity arising in the practice after the outreach meeting? Did you see an action plan during or after this meeting? Did you personally have any tasks assigned to you as a result of taking part in ASPIRE? If so, what has happened since? If not, who has been doing work as a result of ASPIRE? How was this decided? What do you think of the way this has been done?

*Implementation*

To your knowledge, has anything in the practice been done differently as a result of involvement in ASPIRE?

If yes, explore what has been done differently, who is involved in the changes and what the intentions are behind the changes

Explore positives and negatives of changes—expected changes, unexpected changes, ones that seem to be benefitting practice, staff and/or patients, ones that do not or where they cannot value impact yet

Are you aware of any plans to do anything differently in the practice as a result of ASPIRE?

If yes, explore what plans there are, who is involved in them and how, and what the intentions/goals are behind these plans

Explore progress on the plans—how far has this gotten? When are things likely to happen? What’s affecting the timing of the plans?

Has involvement in ASPIRE affected you or your work in any ways? If so, how? Has it had any impact in the practice in general or any impact on anyone else (that you are aware of)?

Drill into specific details

*Usefulness and fit*

In your opinion, can you think of any specific ways that ASPIRE has been useful for the practice? Who do you think has benefitted (and how)?

Is there anything you think would have been helpful, but has not been offered or done as part of involvement in ASPIRE?

Was this intervention a good fit for your practice? If so, in what ways? If not, why not? What do you think did not fit with the practice? What in particular worked or did not work for your practice?

Probe around clinical topic, intervention components, targets for intervention (e.g. practitioner behaviour and practice organisation)

*Close*

Who else in the practice do you think would be good to talk to?

### Appendix 2 Observational guide

General Practice—Organisation and Setting

Organisation of care—general

Setting—physical space, frequency and nature of meetings, size of practice

Practice group dynamics—no. of staff, relations between them, decision-making

Other initiatives on the agenda—related to clinical topic/unrelated but potential to

impact

ASPIRE related

Responses to and expectations of A&F form

Responses to and expectations of educational outreach

Responses to and expectations of the searches

Responses to and expectations of the prompts

Responses to and expectations of offer of help

Interactions around set up of meetings

Questions relating to the research

Responses to and expectations of other ASPIRE elements (checklist, prompt, pens/post

its/laminates, SEA forms)

My role and relation to practices

Understandings of/reactions to my role

Clinical topic

Organisation of care specific to practice topic

Work done by practice or others for practice on clinical topic

Responses to clinical topic

Perceived barriers or areas of concerns or needs of the practice, including patient,

practice and system factors (domains, CMO)

Reasons for/discussion of lack of need or concern around this topic

Questions relating to the clinical topic

Patient cases or examples discussed

### Appendix 3 Interview guide for final practice meeting

How you feel you did as a practice in relation to the topic area

*Move conversation along quickly*

*How interested was the practice in this topic before the year started*? *Did ASPIRE stimulate an interest in improving in this area?*

What helped or didn’t help over the year with regards to the topic area

*What helped from ASPIRE, what helped generally*

*What didn’t help*

What effects, if any, the various ASPIRE intervention components have had over the year, and what they have meant to people in the practice

Audit and feedback reports

Outreach visits (1 or 2)

Outreach support

Searches

Prompts (risky prescribing only)

Laminates and pens/post its

Taking part in the process evaluation

What could be done differently in the future

How research can support primary care to implement research evidence

What the practice themselves could do to improve implementation

Role of CCGs, federations, network

What, if anything, the practice intends to do next with regards to work on the specific clinical topic

## Appendix 4 Normalisation Process Theory (NPT) coding dictionary

Tables 9 and 10

**Table 9:** NPT—understanding the process of implementation within practices

NPT constructs	Definition	Application
**Coherence**	**Making the practice coherent**	
Differentiation	Does the practice differ from other work?	Indications from observation/interviews that the intervention (by component or whole) is differentiated from other work (conversely, indications that it is indistinct from other work)
Communal specification	Does the practice make sense to the group?	Examples of sense-making in group settings (or individual reflections on group sense-making) around what the intervention entails in terms of actions and consequences
Individual specification	Does the practice make sense to the individual?	Examples of sense-making at the individual level around what the intervention entails in terms of actions and consequences; also, instances where different individuals make sense of the intervention in differing ways
Internalisation	Does the practice link to personal norms and values?	Any links made by individuals regarding their personal/professional norms and values and how they align (or not) to what the intervention entails in terms of actions and consequences; also, any inferable links observed in meeting discussions
**Cognitive Participation**	**Enrolling people into the practice**	
Legitimisation	Do they work together to produce agreement?	Indications from observation/interviews that the team has discussed the intervention and its implementation in the practice, looking for who was involved in discussions and whether or not people see things in the same way
Enrolment	Do they find ways to work together to engage in practice?	Indications from observation/interviews that the team has considered in practical terms how to implement the intervention, and looking for what plans (if any) were developed and who these plans involved. Consider how concrete and detailed these plans were and whether the individuals identified to act were involved in the assignment of tasks
Initiation	Do they initiate the practice in specific times and places, with resources?	Indications from observation/interviews as to whether people have acted as a result of intervention (whole or components), and when and how they have acted. Consider who the actors were, and what resources were required to act. Also consider plans to act which failed (and why they may have failed)
Activation	Do they collectively work out ways to sustain the practice over time?	Indications from observation/interviews that people within the practice have considered the maintenance of any actions as a result of the intervention or are sustaining work over time in any way; also, consider where they have the intent to sustain but fail to do so
**Collective Action**	**Enacting the practice within context**	
Contextual integration	Do they work to realise necessary resources and direct them in support of the practice?	Consider how the practice or individuals involved allocate resources and whether this changes as a result of the intervention; consider how their decisions around resources might impact on uptake of the intervention (whole or components)
Relational integration	Do they do knowledge work to build accountability and maintain confidence in the practice and each other?	Consider how the practice or individuals within the practice make sense of the intervention in relation to each person’s responsibilities and capabilities to complete the actions implied and how the team works (or doesn’t) in order to achieve shared goals. Attend to points of weakness, where people lack confidence in the intervention or each other
Interactional workability	Do they develop ways to work with each other and other resources to accomplish the practice?	Following on from enrolment in the work, are there indications that the practice and individuals within the practice are building on their initial plans of work, making amendments where necessary or adjusting practices/resource allocations in order to achieve shared goals relating to the intervention.
Skill-set workability	Have they divided the labour out and know who will do what and how to accomplish the practice?	Consider from observation/interviews, how the practice has divided labour and how well defined their plans are as to who does what. Consider how this is communicated, and who is involved in decisions, and how well it suits understandings (individually and collectively) of people’s skills and capabilities
**Reflexive monitoring**	**Monitoring and sustaining the practice over time**	
Systematisation	Do they work out a system to define, collect and collate information about effects?	Consider what the practice or individuals therein may decide to do to track progress—does it involve intervention components, or practice-developed strategies, or both? Consider failures to do this as well and where the failures may occur
Communal appraisal	Do they work together to evaluate the worth of the practice?	Consider any evidence of communal appraisal of the intervention and actions implied by the intervention—what is the group narrative around the value of the intervention?
Individual appraisal	Do they appraise the practice from their own experience?	Consider any evidence of individual appraisal of the intervention and actions implied by the intervention—what individual narratives are identifiable around the value of the intervention, and do they match any communal narratives?
Reconfiguration	Do they do any work to redefine or modify practice?	Consider whether the practice or individuals therein have amended or redefined the actions undertaken as a result of the intervention over time, and any rationales for changes

**Table 10:** Theoretical Domains Framework (TDF) coding dictionary. TDF—understanding the factors which impede or promote intervention behaviours or action [10]

TDF—domains	Definition	Application
Knowledge	An awareness of the existence of something Possible sub-domains: knowledge; procedural knowledge; knowledge of task environment	Are there any indications in observation/interviews that knowledge of the clinical topics or what to do to improve outcomes is acting as a barrier to achievement? Any indications that there is either a lack of knowledge or conversely, that knowledge is *not* an issue? *Core analytic question: What role does knowledge play in the adoption or maintenance of behaviours relating to the intervention or the clinical topic?*
Skills	An ability or proficiency acquired through practice Possible sub-domains: skills; skills development; competence; ability; interpersonal skills; practice; skill assessment	Are there any indications in observation/interviews that skills around the behaviours attached to the intervention are affecting change? *Core analytic question: What role do skills play in the adoption or maintenance of behaviours relating to the intervention or the clinical topic?*
Social/Professional Role and identity	A coherent set of behaviours and displayed personal qualities of an individual in a social or work setting Possible sub-domains: professional identity; professional role; social identity; identity; professional boundaries; professional confidence; group identity; leadership; organisational commitment	Are there any indications in observation/interviews that the roles or identities of the practitioners/staff are influencing adoption or maintenance of behaviours attached to the intervention? *Core analytic question: What role does social and professional roles play in the adoption or maintenance of behaviours relating to the intervention or the clinical topic?*
Beliefs about capabilities	Acceptance of the truth, reality, or validity about an ability, talent, or facility that a person can put to constructive use Possible sub-domains: self-confidence; perceived competence; self-efficacy; perceived behavioural control; beliefs; self-esteem; empowerment; professional confidence	Are there any indications in observation/interviews that beliefs about capabilities are influencing adoption or maintenance of behaviours attached to the intervention? Beliefs about capabilities may be individual, about others, and about the system *Core analytic question: What role do beliefs about capabilities play in the adoption or maintenance of behaviours relating to the intervention or the clinical topic?*
Optimism	The confidence that things will happen for the best or that desired goals will be attained Possible sub-domains: optimism; pessimism; unrealistic optimism; identity	Are there any indications in observation/interviews that confidence levels regarding the intervention and the likelihood of success are influencing adoption or maintenance of behaviours attached to the intervention? *Core analytic question: What role does optimism play in the adoption or maintenance of behaviours relating to the intervention or the clinical topic?*
Beliefs about consequences	Acceptance of the truth, reality, or validity about outcomes of a behaviour in a given situation Possible sub-domains: beliefs; outcome expectancies; characteristics of outcome expectancies; anticipated regret; consequents	Are there any indications in observation/interviews that beliefs about consequences are influencing adoption or maintenance of behaviours attached to the intervention? *Core analytic question: What role do beliefs about consequences play in the adoption or maintenance of behaviours relating to the intervention or the clinical topic*?
Reinforcement	Increasing the probability of a response by arranging a dependent relationship, or contingency, between the response and a given stimulus Possible sub-domains: rewards; incentives; punishment; consequents; reinforcement; contingencies; sanctions	Are there any indications in observation/interviews that incentives/disincentives are influencing adoption or maintenance of behaviours attached to the intervention? *Core analytic question: What role does reinforcement (reward and punishment) play in the adoption or maintenance of behaviours relating to the intervention or the clinical topic*?
Intentions	A conscious decision to perform a behaviour or a resolve to act in a certain way Possible sub-domains: stability of intentions; stages of change model; transtheoretical model and stages of change	Are there any indications in observation/interviews that motivations and intentions of practitioners/staff are influencing adoption or maintenance of behaviours attached to the intervention? *Core analytic question: What role does motivation/intention play in the adoption or maintenance of behaviours relating to the intervention or the clinical topic*?
Goals	Mental representations of outcomes or end states that an individual wants to achieve Possible sub-domains: goals (proximal/distal); goal priority; goal/target setting; goals (autonomous/controlled); action planning; implementation intention	Are there any indications in observation/interviews that goals of practitioners/staff are influencing adoption or maintenance of behaviours attached to the intervention? *Core analytic question: What role do goals and goal-setting play in the adoption or maintenance of behaviours relating to the intervention or the clinical topic*?
Memory, attention and decision processes	The ability to retain information, focus selectively on aspects of the environment and choose between two or more alternatives Possible sub-domains: memory; attention; attention control; decision making; cognitive overload/tiredness	Are there any indications in observation/interviews that memory, attention or decision-making processes are influencing adoption or maintenance of behaviours attached to the intervention? *Core analytic question: What role does memory, attention, and/or decisional processes play in the adoption or maintenance of behaviours relating to the intervention or the clinical topic?*
Environmental context and resources	Any circumstance of a person’s situation or environment that discourages or encourages the development of skills and abilities, independence, social competence, and adaptive behaviour Possible sub-domains: Environmental stressors; resources/material resources; organisational culture/climate; salient events/critical incidents; person x environment interaction; barriers and facilitators	Are there any indications in observation/interviews that environmental factors are influencing adoption or maintenance of behaviours attached to the intervention? For instance, lack of resources, external pressures, inadequacy of equipment, or vice versa? *Core analytic question: What role does the context and the resources within the practice play in the adoption or maintenance of behaviours relating to the intervention or the clinical topic*?
Social influences	Those interpersonal processes that can cause individuals to change their thoughts, feelings, or behaviours Possible sub-domains: social pressure; social norms; group conformity; social comparisons; group norms; social support; power; intergroup conflict; alienation; group identity; modelling	Are there any indications in observation/interviews that social factors and interpersonal dynamics/processes are influencing adoption or maintenance of behaviours attached to the intervention? *Core analytic question: What role do social factors and interpersonal dynamics play in the adoption or maintenance of behaviours relating to the intervention or the clinical topic*?
Emotion	A complex reaction pattern, involving experiential, behavioural, and physiological elements, by which the individual attempts to deal with a personally significant matter or event Possible sub-domains: fear; anxiety; affect; stress; depression; positive/negative affect; burn-out	Are there any indications in observation/interviews that emotional factors are influencing adoption or maintenance of behaviours attached to the intervention? *Core analytic question: What role does emotion play in the adoption or maintenance of behaviours relating to the intervention or the clinical topic*?
Behavioural regulation	Anything aimed at managing or changing objectively observed or measured actions Possible sub-domains: self-monitoring; breaking habit; action planning	Are there any indications in observation/interviews that issues around behavioural regulation are influencing adoption or maintenance of behaviours attached to the intervention? *Core analytic question: What role does behavioural regulation play in the adoption or maintenance of behaviours relating to the intervention or the clinical topic*?
